# Endothelial Caveolin-1 regulates the radiation response of epithelial prostate tumors

**DOI:** 10.1038/oncsis.2015.9

**Published:** 2015-05-18

**Authors:** D Klein, T Schmitz, V Verhelst, A Panic, M Schenck, H Reis, M Drab, A Sak, C Herskind, P Maier, V Jendrossek

**Affiliations:** 1 Department of Molecular Cell Biology, Institute of Cell Biology (Cancer Research), University of Duisburg-Essen, University Hospital Essen, Essen, Germany; 2 Department of Urology and Urooncology, University of Duisburg-Essen, University Hospital Essen, Essen, Germany; 3 Institute of Pathology, University of Duisburg-Essen, University Hospital, Essen, Germany; 4 Institute of Immunology and Experimental Therapy, Wroclaw, Poland; 5 Wroclaw Research Center EIT+, Wroclaw, Poland; 6 Department of Radiotherapy, University of Duisburg-Essen, University Hospital, Essen, Germany; 7 Department of Radiation Oncology, Universitätsmedizin Mannheim, Medical Faculty Mannheim, Heidelberg University, Mannheim, Germany

## Abstract

The membrane protein caveolin-1 (Cav1) recently emerged as a novel oncogene involved in prostate cancer progression with opposed regulation in epithelial tumor cells and the tumor stroma. Here we examined the role of stromal Cav1 for growth and radiation response of MPR31-4 prostate cancer xenograft tumors using Cav1-deficient C57Bl/6 mice. Syngeneic MPR31-4 tumors grew faster when implanted into Cav1-deficient mice. Increased tumor growth on Cav1-deficient mice was linked to decreased integration of smooth muscle cells into the wall of newly formed blood vessels and thus with a less stabilized vessel phenotype compared with tumors from Cav1 wild-type animals. However, tumor growth delay of MPR31-4 tumors grown on Cav1 knockout mice to a single high-dose irradiation with 20 Gray was more pronounced compared with tumors grown on wild-type mice. Increased radiation-induced tumor growth delay in Cav1-deficient mice was associated with an increased endothelial cell apoptosis. *In vitro* studies using cultured endothelial cells (ECs) confirmed that the loss of Cav1 expression increases sensitivity of ECs to radiation-induced apoptosis and reduces their clonogenic survival after irradiation. Immunohistochemical analysis of human tissue specimen further revealed that although Cav1 expression is mostly reduced in the tumor stroma of advanced and metastatic prostate cancer, the vascular compartment still expresses high levels of Cav1. In conclusion, the radiation response of MPR31-4 prostate tumors is critically regulated by Cav1 expression in the tumor vasculature. Thus, Cav1 might be a promising therapeutic target for combinatorial therapies to counteract radiation resistance of prostate cancer at the level of the tumor vasculature.

## Introduction

Prostate cancer is the most commonly diagnosed malignancy and the second leading cause of death in men worldwide^1^ and its treatment differs depending on patient's age, stage and grade of the tumor.^2^ The grade of differentiation of acinar adenocarcinomas of the prostate is expressed by the Gleason score, which is a sum of the primary and secondary Gleason patterns in resection specimens.^3, 4^ Radical prostatectomy, hormone ablation therapy, percutaneous radiotherapy and interstitial radiation methods are available for the treatment of localized stages yielding >50% of local control.^5, 6, 7, 8^ Radiotherapy is also an integral part of the treatment protocols for inoperable locally advanced prostate cancer. However, resistance to chemotherapy and radiotherapy remains a major obstacle in the successful treatment of high-risk prostate cancer patients. Thus, despite the use of classical chemotherapy (mainly taxanes), hormone ablation therapy, radiopharmaceuticals and refined radiation methods such as intensity-modulated radiation therapy allowing the delivery of increased radiation doses, no curative treatment for advanced stages is available to date. Thus, novel therapy approaches are needed particularly for patients with hormone-refractory disease.^9, 10, 11, 12^

Up to now, agents inhibiting the proliferation or inducing cell death in cancer cells have been the major focus for the development of such anticancer drugs. However, it is now widely accepted that a reactive tumor stroma significantly contributes to growth and malignant progression in prostate cancer.^13, 14, 15^ Increasing evidence further indicates that the heterogeneous tumor stroma supports therapy resistance at multiple levels.^16, 17, 18^ Thus, the identification of molecules and pathways driving stroma-mediated resistance at advanced tumor stages may provide a molecular basis for the development of novel and effective strategies suited to overcome therapy resistance and improve the treatment outcome. Herein, the stroma-derived tumor vasculature attracted major attention for the development of new anticancer drugs.^19, 20^ Interestingly, numerous reports implicate microvascular sensitivity to ionizing radiation in the tumor response to radiation therapy.^21, 22, 23, 24^

The membrane protein caveolin-1 (Cav1) has recently been identified as a marker protein for prostate cancer progression.^25, 26, 27, 28^ Cav1 is a major structural protein that is essential to the formation of caveolae and is predominantly expressed in cells of the stromal compartment, that is, adipocytes, vascular smooth muscle, endothelial cells (ECs) and fibroblasts.^29^ The overexpression of Cav1 in prostate cancer cells, however, had been associated with increased resistance to chemotherapy, metastatic disease and poor prognosis.^30, 31, 32, 33, 34, 35^ Moreover, patients with advanced prostate cancer had increased serum levels of Cav1, suggesting a secretion of Cav1 from prostate cancer cells that may contribute to the tumor-promoting effects of Cav1.^36, 37, 38^ Of note, though levels of Cav1 increased in epithelial cancer cells during prostate cancer progression, Cav1 expression was decreased in the tumor stroma in advanced and metastatic prostate cancer tissue specimen.^25^ Importantly, the loss of Cav1 in prostate cancer stroma was found to be functionally relevant to tumor progression and to correlate with reduced relapse-free survival.^39^

Studies in other cancer types have implicated Cav1 as a pro-survival factor mediating resistance of pancreatic and lymphoblastoid cancer cells to the cytotoxic action of ionizing radiation *in vitro*. Silencing of Cav1 in pancreatic cancer cell lines resulted in the disruption of its interactions with beta1-integrin and focal adhesion kinase leading to reduced cell adhesion, proliferation and survival after exposure to ionizing radiation.^40, 41, 42^ Similarly, Cav1 expression also protected lymphoblastoid TK6 from radiation-induced apoptosis.^43^

Here we investigated the role of stromal Cav1 for growth- and resistance-promoting tumor-stroma interactions during prostate cancer progression with a focus in the vascular compartment. For this, we used MPR31-4 murine prostate cancer xenografts growing on Cav1-proficient and Cav1-deficient C57Bl6 mice.^44^ We show that the recruitment of smooth muscle actin-positive cells to tumor microvessels is defective in Cav1-deficient mice with potential relevance for increased tumor growth. Moreover, we demonstrate that endothelial Cav1 is a critical regulator of microvascular sensitivity to ionizing radiation in MPR31-4 prostate cancer xenograft tumors with impact on tumor growth delay after local irradiation. Lowering Cav1 expression in cultured ECs facilitated radiation-induced apoptosis and reduced clonogenic cell survival of endothelial cell after irradiation *in vitro*. Our findings suggest a potential use of the pro-survival protein Cav1 as a therapeutic target for overcoming therapy resistance in prostate cancer.

## Results

### Single high-dose irradiation decreased growth of MPR31-4 xenograft tumors more efficiently in Cav1-deficient mice

**Figure 1:**
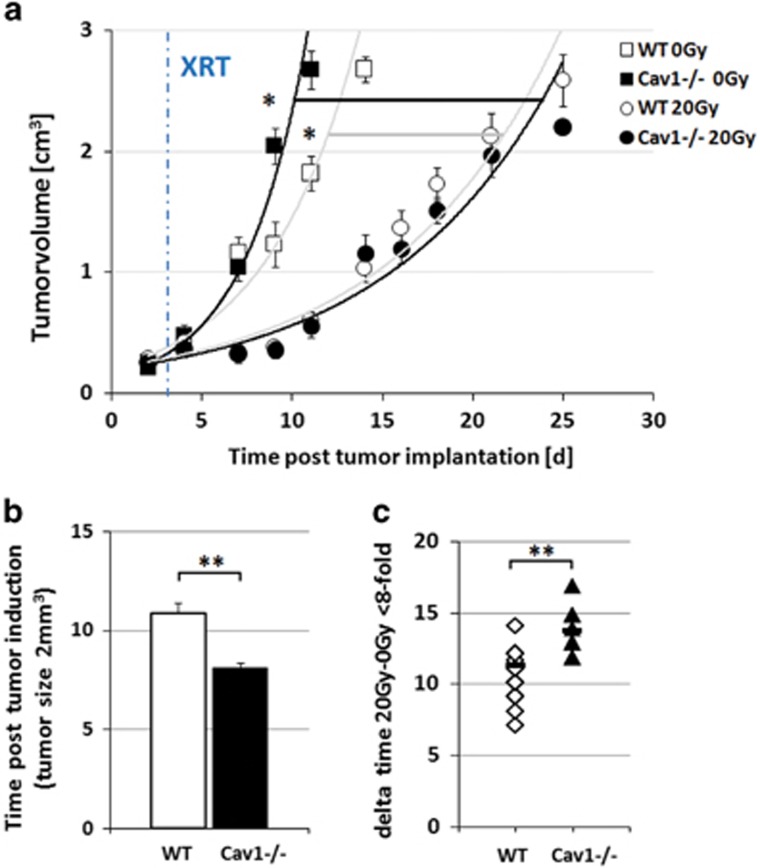
Single-dose irradiation decrease growth of MPR31-4 xenograft tumors more efficiently when tumors were grown on Cav1-deficient mice. (**a**) MPR31-4 tumor cells (0.5 × 10^6^ cells each) were subcutaneously transplanted onto the hindlimb of Cav1-deficient mice (knockout (KO), black squares) and wild-type littermates (WT, white squares). One set of animals from each strain received a single radiation dose of 20 Gy to the tumor after manifestation of the tumor at day 4 (KO, black circles; WT, white circles). Tumor volume was determined at indicated time points using a sliding caliper. Data are presented as mean±s.e.m. from three independent experiments (49 mice in total). WT (*n*=14), KO (*n*=13), WT 20 Gy (*n*=13), KO 20 Gy (*n*=9). (**b**) Tumor growth was determined as time (days) until a tumor volume of 2 cm^3^ was reached WT (*n*=14), KO (*n*=9). (**c**) Respective computed median growth delay was determined as time (days) until the eightfold tumor volume was reached. WT 20 Gy (*n*=13), KO 20 Gy (*n*=9). *P<0.05; **P<0.01.

To examine the role of stromal Cav1 in prostate tumor radiosensitivity, we first compared the response of MPR31-4 tumor xenografts to a single high-dose irradiation in Cav1 wild-type and Cav1-deficient mice (Figure 1). For this, subcutaneous MPR31-4 prostate xenografts were implanted into Cav1-deficient mice and their wild-type littermates and were irradiated locally with a single dose of 20 Gray (Gy) when the tumor reached a size of about 100 mm^3^ (around day 4). Tumor growth was determined by measuring the tumor volume three times a week (Figure 1a). Tumors implanted into Cav1-deficient mice showed a significantly increased tumor growth when compared with the tumors grown in the wild-type controls (Figures 1a and b). Moreover, tumor growth delay after radiation was significantly increased in Cav1-deficient mice (Figures 1a and c).

### Prostate tumors grown in Cav1-deficient mice displayed a less stabilized vascular phenotype

**Figure 2:**
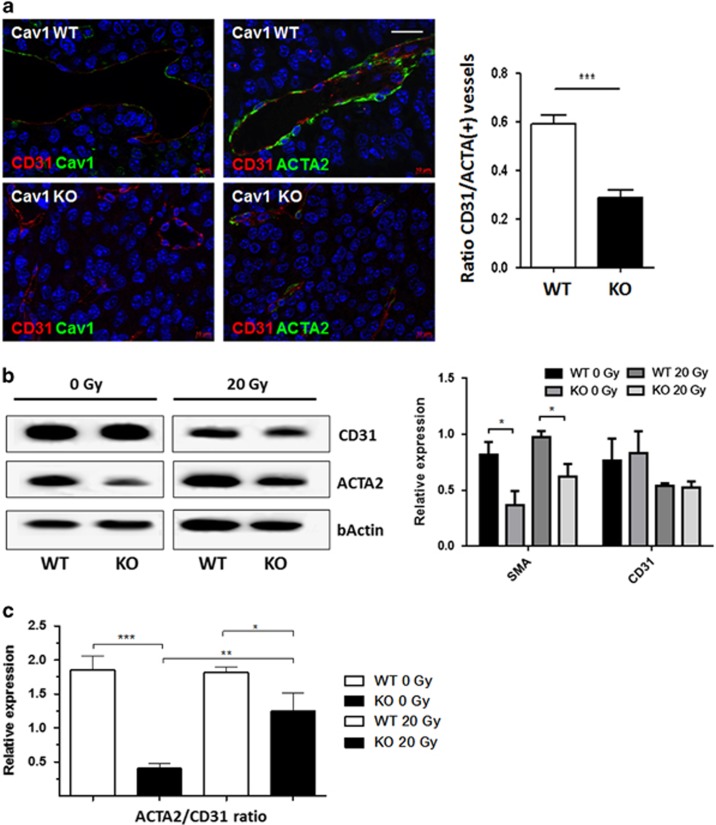
Prostate tumors grown in Cav1-deficient mice showed a less stabilized vascular phenotype. (**a**) Subcutaneously grown MPR31-4 tumors were analyzed by immunofluorescence and confocal microscopy. Left panel: vessels were stained for CD31 (ECs, red) and Cav1 (green) or smooth muscle actin (ACTA2; pericytes/smooth muscle cell, green). Scale bar. 50 μm. Right panel: CD31- and ACTA2-positive blood vessels were quantified by counting immunoreactive structures in whole-tissue sections and the ACTA2/CD31 ratio was calculated (*n*=5). (**b**) CD31 and ACTA2 expression levels were determined using western blot analysis. (**c**) Protein levels were analyzed by densitometry and normalized to β-actin (bActin) levels (relative protein expression). Data are presented as means±s.d. from three independent experiments with multiple samples (*n*=5 per group).

In order to investigate whether the differences in the vascular compartment between tumors grown on Cav1-deficient and Cav1-proficient mice may contribute to the observed findings, subcutaneously grown MPR31-4-tumors were analyzed by immunofluorescence in combination with confocal microscopy (Figure 2). Interestingly, in wild-type controls smooth muscle actin (ACTA2)-positive cells were regularly and closely associated with newly formed Cav1-expressing CD31(+) blood vessels, whereas Cav1-deficient vessels of tumors grown in Cav1-deficient mice lacked smooth muscle cell elements in their wall indicating less stabilized and thus less mature blood vessels (Figure 2a, left panel). Quantification of CD31- and ACTA2-positive vessels by counting immunoreactive structures confirmed that tumors grown in Cav1-deficient animals have less vessels stabilized by ACTA2(+) smooth muscle cells (Figure 2a, right panel). Ultrastructural analysis revealed an irregular lining of smooth muscle cells and the presence of fenestrae in angiogenic ECs from tumors of Cav1-deficient animals, thereby corroborating the less mature and functional inferior phenotype of these tumor vessels (Supplementary Figure S1). Quantification of endothelial (CD31) and ACTA2 protein expression using western blot analysis further endorsed that vessels of tumors grown in Cav1-deficient animals have less ACTA2 indicating less vascular stabilization (Figures 2b and c). Interestingly ACTA2 levels in MPR31-4 tumors grown in Cav1-deficient mice increased after irradiation resulting in a higher ACTA2/CD31 ratio that may be indicative for increased vessel stabilization, in particular a temporally delayed stabilization (Figures 2b and c).

### Radiation therapy reduced Cav1 expression in MPR31-4 tumors and increased caspase 3 cleavage in tumors grown in Cav1-deficient animals

**Figure 3:**
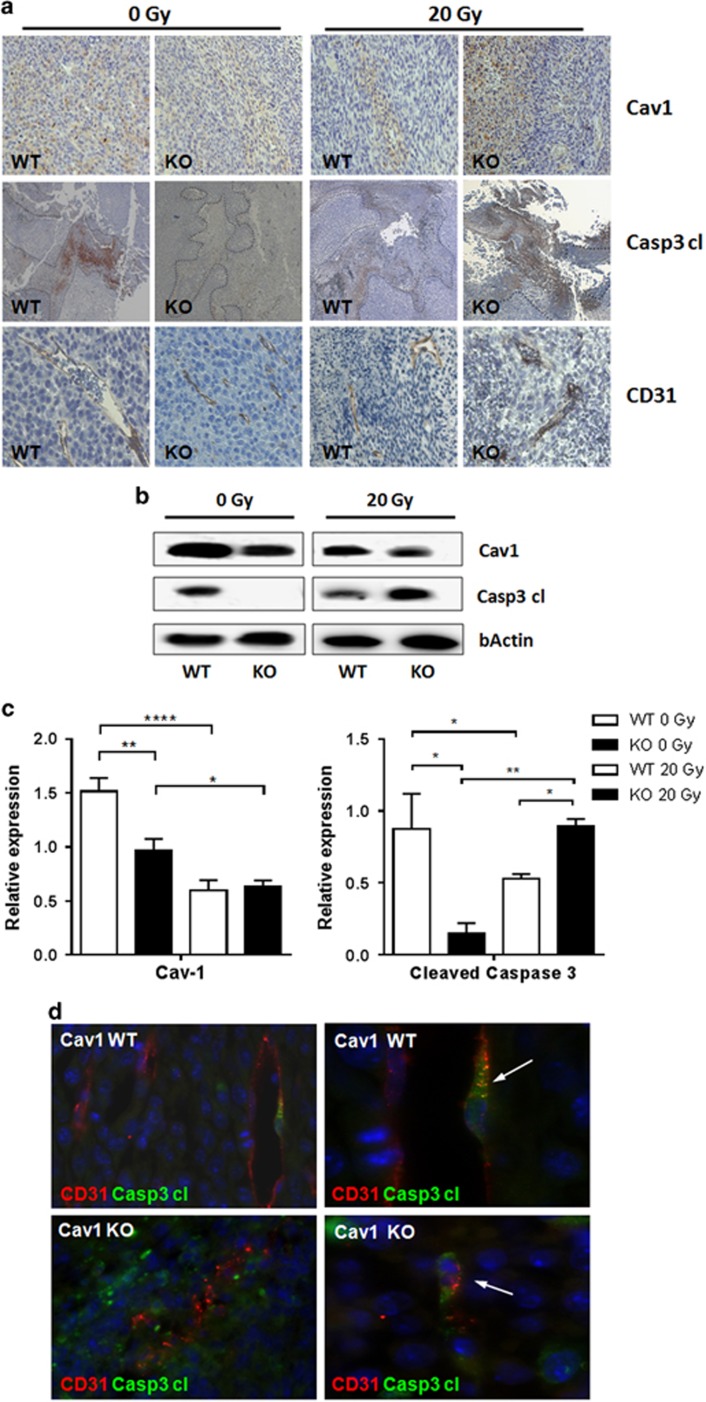
Radiation therapy induced increased caspase 3 cleavage in MPR31-4 tumors and tumor endothelial cell apoptosis in Cav1-deficient animals. (**a**) MPR31-4 tumor cells were subcutaneously transplanted into Cav1-deficient mice (knockout (KO)) and wild-type controls (WT). When tumor volumes reached a critical size (5–20 days after tumor irradiation) tumors were removed subjected to immunohistochemistry. Sections were stained for Cav1, cleaved caspase 3 (Casp3 cl) and CD31 proteins. Dotted lines emphasize the border necrotic and normal tumor tissue. (**b**) Levels of Cav1 and Casp3 cl were further determined using western blot analysis. Representative blots from at least three independent experiments are shown. (**c**) Protein levels were analyzed by densitometry and normalized to β-actin (bActin) levels (relative protein expression). Data show representative blots from at least three independent experiments. (**d**) MPR31-4 tissue sections of irradiated tumors were stained for CD31 (red) and Casp3 cl (green) or smooth muscle actin (ACTA2; pericytes/smooth muscle cell, green). Arrows point toward CD31-positive ECs, which became immunoreactive for Casp3 cl upon irradiation (4 days post XRT (post-operative radiotherapy)). Representative images from at least three independent experiments are shown. Magnification × 63.

The above data suggested that the destabilized Cav1-deficient vessels are more sensitive to ionizing radiation. Consistent with this radiation therapy resulted in the increased cleavage of Casp3 (Caspase 3) in tumors grown in Cav1-deficient animals (Figures 3a–c). Herein, viable tumor cells were arranged close to the tumor blood vessels. In Cav1-deficient animals these tumor vessels were smaller in size and showed an impaired morphology after irradiation accompanied by increased areas of necrosis close to the affected angiogeneic vessels (Figure 3a). Moreover, radiation therapy reduced the overall Cav1 expression levels in MPR31-4 tumors grown in Cav1-proficient and Cav1-deficient mice, an effect which may be due to the impaired growth of Cav1-positive tumor cells after radiotherapy (Figures 3a–c). To gain more insight into the relevance of endothelial Cav1 for growth and radiation response of prostate epithelial tumors, we additionally compared tumor hypoxia and tumor cell proliferation in tumors grown on Cav1-proficient and Cav1-deficient mice on the histological level by using co-immunostaining for endothelial CD31 expression and hypoxia inducible factor 1-alpha (HIF1-alpha) and the proliferation marker Ki67, respectively (Supplementary Figure 2). Prostate tumors grown on Cav1-deficient mice were characterized by an increased expression of HIF1-alpha indicative for tissue hypoxia as well as by regions with enhanced cell proliferation compared with the tumors grown on wild-type mice that may contribute to increased growth of tumors in Cav1-deficient mice. A reduction in HIF1-alpha as well as of Ki67 levels after irradiation was detected in tumors grown in Cav1-deficient as well as wild-type mice, yielding more comparable levels of HIF1-alpha and Ki67-positive areas in irradiated tumors (Supplementary Figure 2).

Immunofluorescence analysis further revealed that increased caspase 3-activation was confined to CD31-positive cells indicating a higher sensitivity of Cav1-deficient ECs to radiation-induced apoptosis (Figure 3d). Furthermore vascular structures in areas with increased immunoreactivity of cleaved caspase 3 seemed to be less regular and more degraded, which hints to the contribution of vascular damage to the more pronounced antineoplastic effect of radiotherapy in the Cav1-deficient tumors.

### Reduction of Cav1 levels increases radiation-induced endothelial cell death and decreases the survival of clonogenic ECs *in vitro*

**Figure 4:**
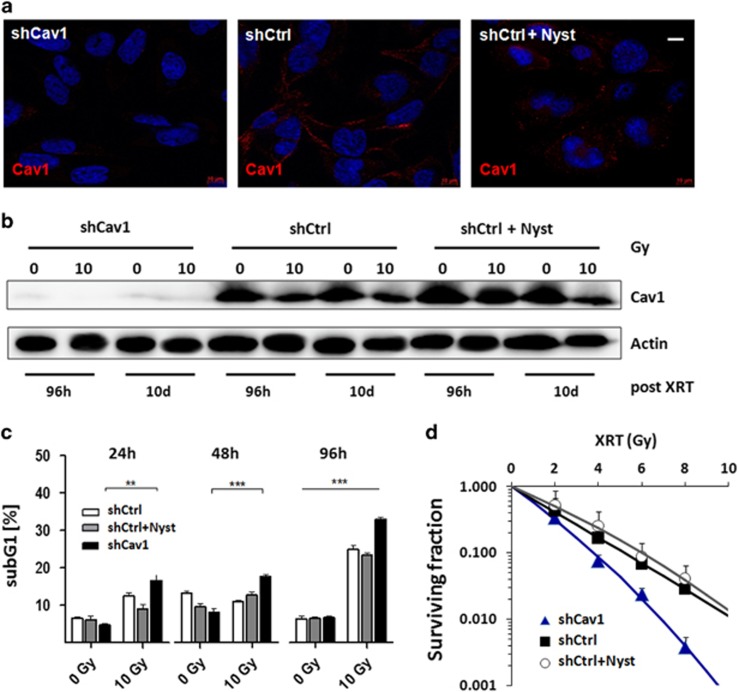
Reduction of Cav1 levels increased radiation-induced endothelial cell death and decreased the survival of clonogenic ECs. (**a**) Cav1 expression and localization was analyzed in cultured shCav1-transfected and control-transfected (shCtrl) AS-M5 ECs by immunofluorescence (red). Nuclei were stained in blue. In addition, control-transfected AS-M5 cells were treated with nystatin (10 μg/ml) in order to inhibit caveolae formation (shCtrl+Nyst). (**b**) Cav1 expression was further determined using western blot analysis. (**c**) The degree of apoptosis was quantified by measuring the SubG1 fraction after radiation by flow cytometry analysis. SubG1 levels were related to the 0 Gy (control) value (set as 1). (**d**) AS-M5 cells were plated for colony formation assay, irradiated with indicated doses and subsequently further incubated for additional 10 days. Data show the surviving fractions from three independent experiments (means±s.d.).

To confirm our hypothesis that ECs with reduced Cav1 expression are more sensitive to ionizing radiation, we performed *in vitro* experiments using the endothelial cell line AS-M5 in combination with shRNA knockdown of Cav1 expression (Figure 4). In order to further analyze whether a reduced Cav1 expression or the disruption of Cav1-dependent caveolae might have been responsible for the observed increased endothelial cell apoptosis upon radiation control-transfected AS-M5 cells were treated with nystatin, a well known chemical inhibiting caveolae formation (shCtrl+Nyst).

Lentiviral expression of a Cav1-specific siRNA in AS-M4 ECs (AS-M5 shCav1) resulted in an efficient and sustained downregulation of Cav1 expression compared with the control-transduced (shCtrl) cells as shown by immunofluorescence and western blot analysis, respectively (Figures 4a and b). In contrast nystatin-treatment did neither affect Cav1 content nor its localization in the cells (Figures 4a and b and Supplementary Figure S2). After treatment Cav1 is still mainly localized to the plasma membrane as demonstrated by western blot analysis, whereas the local concentration at the plasma membrane seemed to be more diffuse as indicated by an less intensive plasma membrane staining in the immunofluorescence analysis (Figure 4a and Supplementary Figure S2). Electron microscopic analysis further confirmed that nystatin-treatment resulted in the disruption of caveolea (Supplementary Figure S2). Instead, endothelial Cav1 expression levels were reduced in shCtrl cells and in nystatin-treated shCtrl cells after exposure to ionizing radiation (Figure 4b). Interestingly, the reduction of Cav1 levels decreased endothelial cell proliferation but did not affect the cell cycle distribution (Supplementary Figure S4). Of note, the reduction of the Cav1 protein level resulted in an increased sensitivity of ECs to radiation-induced apoptosis as shown by the pronounced increase in the sub-G1-fraction (Figure 4c). In line with these findings, a long-term assay measuring the surviving fraction after irradiation revealed that the number of ECs able to regrow and form a colony after irradiation was considerably diminished in AS-M5 shCav1 cells compared with the AS-M5 shCtrl cells with normal Cav1 expression (Figure 4d). Instead, nystatin-treatment did neither result in an increased sensitivity of AS-M5 shCtrl cells to radiation-induced apoptosis nor in a more pronounced eradication of clonogenic AS-M5 cells (Figures 4c and d). These findings indicate that indeed the reduced Cav1 expression and not the disruption of caveolar structures is responsible for the observed radiosensitization of AS-M5 shCav1 cells. On the basis of these observations we speculate that the loss of Cav1 in ECs might be responsible for the more pronounced tumor growth delay upon irradiation of MPR31-4 tumors grown on mice with stromal Cav1-deficiency. Consequently lowering Cav1 levels in tumor ECs may be suited to improve the outcome of radiation therapy in prostate cancer.

### Loss of stromal Cav1 in advanced prostate cancer does not extend to the vasculature

**Figure 5:**
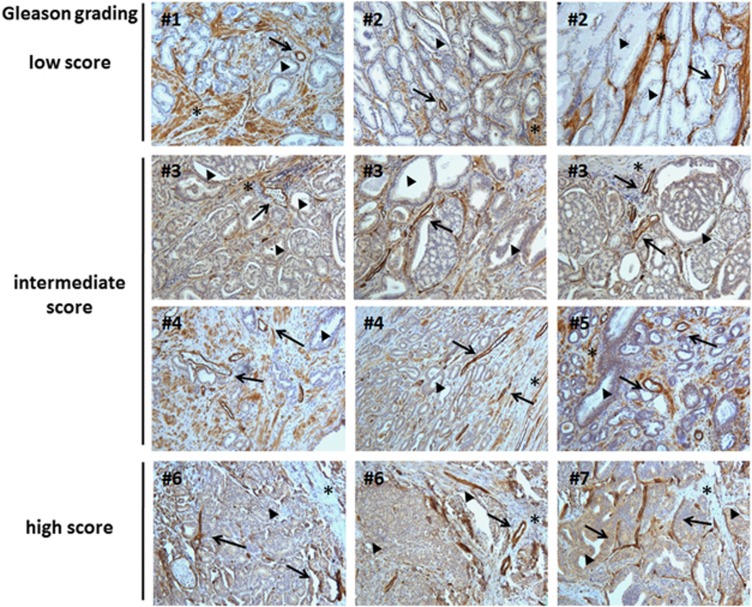
Immunohistological analysis of Cav1 expression in human prostate tumor tissues. Paraffin sections of human prostate tumors were stained for Cav1. Gleason grading scores, used to evaluate the prognosis of men with prostate cancer, were divided into low (1+1, 2+2), intermediate (3+3, 4+3) and high scores (4+5) according to the sum of the primary and secondary Gleason patterns in whole-resection specimens. The observed patterns of the tumor specimen were assigned based on the current WHO (World Health Organization) and the updated ISUP criteria: the primary grade—assigned to the dominant pattern of the tumor (has to be >50% of the total pattern seen) as well as a secondary grade—assigned to the next-most frequent pattern (has to be <50%, but at least 5%, of the pattern of the total cancer observed). The actual Gleason score of the photographs shown is 2 for each photograph of the low, 3 for intermediate and 4 for high Gleason scores. Asterisks mark stromal compartments, bold arrowheads point to epithelial structures and arrows emphasize Cav1-positive vessels. Sections were counterstained using hematoxylin. Representative images of nine tissue samples are shown. # indicates different patients and panels of the same patient's number, show different areas of the same resection specimens. Magnification × 20.

To evaluate if Cav1 is also highly expressed in ECs of the tumor stroma in human prostate cancer tissue, which is a prerequisite for its use as potential therapeutic target, human prostate tissue specimens were analyzed for endothelial Cav1 expression. Therefore, formalin-fixed paraffin-embedded tissue slides of human prostate adenocarcinomas with distinct Gleason scores were immunostained for Cav1 (Figure 5). In line with the previous reports, we found that benign prostate epithelia were negative for Cav1 but that Cav1 expression in prostate epithelial cells increased with higher Gleason scores, that is, lower tumor differentiation (Figure 5, bold arrows). In contrast, stromal cells of tumor samples tended to be less intensively stained or even negative in cases with higher Gleason grade (Figure 5, asterisks). However, ECs in tumor vessels did not exhibit an altered Cav1 expression during prostate cancer progression: a high expression of Cav1 was detected in the tumor vasculature in all tissue samples analyzed independently from the Gleason score (Figure 5 arrows). The analysis of different areas of the same resection specimen revealed that the maintenance of endothelial Cav1 expression in advanced prostate tumors is not restricted to certain tumor areas with a particular Gleason grade but a general observation.

**Figure 6:**
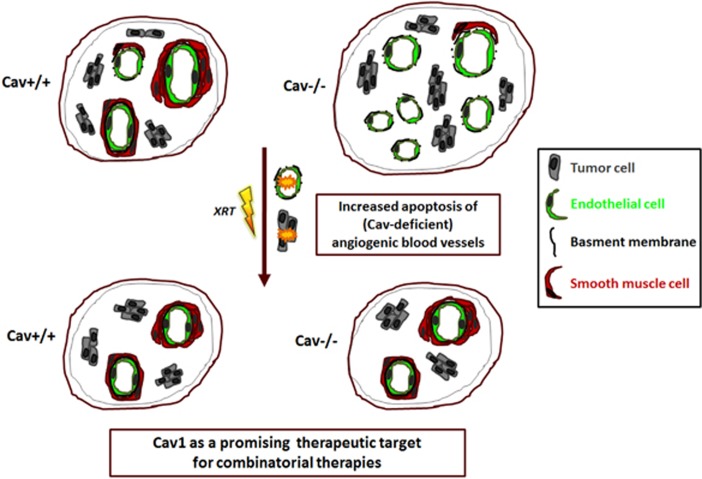
Tumor growth and tumor response to radiation therapy critically depend on endothelial Cav1 expression. Syngeneic MPR xenografts implanted into Cav1-deficient C57Bl/6 mice grew faster and displayed less integration of smooth muscle cells into the wall of newly formed blood vessels indicative for a less stabilized vessel phenotype compared with tumors from Cav1 wild-type animals. High Cav1 expression in ECs protected vascular structures from radiation-induced damage at a clinically relevant dose, resulting in a decreased tumor growth delay upon irradiation. In contrast, the loss of stromal Cav1 increased the sensitivity of ECs to radiation-induced apoptosis thereby enhancing tumor growth delay upon radiotherapy. Thus, Cav1 content of vascular cells determines the sensitivity to microvascular damage and is critical for the regulation of the tumor response to radiation. Thus, the pro-survival factor Cav1 might be a promising therapeutic target for combinatorial therapies to counteract radiation resistance of prostate cancer at the level of the tumor vasculature.

Our data suggest that endothelial Cav1 is a promising therapeutic target to overcome radiation resistance in prostate cancer (Figure 6).

## Discussion

During the last years, interest has been increasing in understanding the role of stromal cells in tumor responses to radiotherapy and chemotherapy. Here we show for the first time that the recruitment, association and integration of smooth muscle cells into the wall of newly formed blood vessels is defective in prostate MPR31-4 tumors grown in Cav1-deficient mice giving rise to vessels of a less stabilized, proangiogenic phenotype that facilitate tumor growth. Importantly, the loss of Cav1 enhanced the sensitivity of microvascular ECs to radiation-induced apoptosis *in vitro* and *in vivo* leading to a more pronounced growth delay of tumors grown in Cav1-deficient animals upon a single high-dose irradiation with 20 Gy. Our data indicate that radiation response of MPR31-4 prostate tumors is critically regulated by Cav1 expression in the tumor vasculature. Thus, Cav1 might be a promising therapeutic target for combinatorial therapies to counteract radiation resistance of prostate cancer at the level of the tumor vasculature.

In more detail, our data on the enhanced growth of MPR31-4 tumors in Cav1-deficient mice corroborate earlier findings on increased growth of Lewis lung carcinoma tumors implanted to Cav1-deficient mice.^45^ The authors of this study attributed the higher growth rates of Lewis lung carcinoma cells on Cav1-deficient mice to increased tumor angiogenesis and decreased tumor cell death. In line with these findings, Dewever *et al.*^46^ showed a higher density of CD31-positive vascular structures and a marked deficit in alpha smooth muscle actin-positive mural cells in tumor specimen from B16 melanoma-bearing Cav1-deficient mice. However, we show here for the first time that Cav1 levels in the tumor microvasculature are important to the outcome of radiotherapy. These observations are of potential clinical relevance in view of the assumption that the heterogeneous tumor stroma may contribute to therapy resistance and thus to poor clinical outcome in cancer patients.

A carcinoma is a complex structure consisting of cancerous cells and stromal cells, including fibroblasts/fibrocytes, pericytes, ECs and immune cells. Under physiological conditions the stroma is an important barrier to malignant transformation of cells. However, the stroma changes its functional role during neoplastic transformation and has a key role in cancer cell invasiveness and progression, and potentially therapy resistance.^47, 48^ Particularly the interest in understanding the role of the microvasculature in normal tissue and tumor responses has shed new light on the critical role of tissue microvasculature in regulating the tumor response to radiation and drugs.^49^ Recent genetic and pharmacologic studies have revealed that the crosstalk between tumor cells and the host-derived tumor microvasculature, both affected by radiation, constitutes a generic element of the pathogenesis of radiation-induced tissue damage.^50, 51, 52, 53^ In prostate cancer androgen-mediated upregulation of vascular endothelial growth factor led to the formation of tumors with a less mature and leaky tumor vasculature.^54^ Thereby it hypothesized that pharmacological inhibition of vascular endothelial growth factor triggered an increased apoptosis of ECs in these immature and leaky blood vessels, leading to the selection for mature, non-leaky vessels the so-called ‘pruning effect'. The resulting increase in oxygenation in turn would then enhance the sensitivity of the tumors to ionizing radiation.^54^

Our data demonstrate that endothelial Cav1 functions as another critical regulator of vascular function and microvascular sensitivity to ionizing radiation with impact on radiation-induced tumor growth delay. Interestingly, tumor-free Cav1-deficient animals are also characterized by a hyper-permeable vasculature that is associated with organ pathologies like cardiomyopathy and pulmonary dysfunction.^44, 45^ Of note, specific reconstitution of Cav1 in ECs led to a normalization of vascular, cardiac and pulmonary phenotypes, demonstrating that the pathologic phenotypes observed in the Cav1-deficient mice are mainly due to the lack of endothelial Cav1 expression.^55, 56^ Likewise we speculate here that the lack of endothelial Cav1 is critical for increased growth of prostate tumors growing on Cav1-deficient mice and that microvascular sensitivity to ionizing radiation is critical to the higher effect of radiotherapy in these tumors. In line with that idea, we show here that prostate tumors grown on Cav1-deficient mice were characterized by an increased tissue hypoxia as well as enhanced cell proliferation compared with tumors grown on wild-type controls. In contrast, the levels of tumor hypoxia and proliferation were more similar in Cav1-deficient and wild-type mice after irradiation. In general, hypoxia was shown to be a strong inducer of angiogenesis that is critical for the development of the malignant prostate.^57, 58^ Within these process the transcription factor HIF-1 alpha is stabilized under hypoxic conditions and induces a number of pathways important in tumor progression, such as angiogenesis, glycolysis and proliferation.^59, 60^ Furthermore, hypoxia and the concurrent increase in tumor cell proliferation in prostate tumors is generally associated with an aggressive phenotype and poor prognosis, although the mechanisms underlying the high proliferation capacity of hypoxia-positive tumors is not completely understood.^61, 62^ Thus, increased tissue hypoxia and tumor cell proliferation in Cav1-deficient mice may contribute to increased tumor growth in these mice compared with the wild-type controls, whereas the more tissue hypoxia and tumor cell proliferation in both mouse strains after irradiation may provide a potential explanation for the similar growth rate of tumors regrowing after irradiation. Nevertheless, further investigations are necessary to corroborate our findings on the role of endothelial Cav1 as critical regulator for the radiation response of epithelial prostate tumors in more clinical relevant settings, such as fractionated low-dose irradiation as well as with more clinically relevant endpoints including local tumor control and tumor recurrence experiments and will be performed in the future.

There is accumulated evidence that the expression of the caveolae-forming protein Cav1 is deregulated in prostate cancer. Although Cav1 has been described as a tumor suppressor gene, the protein has tumor-promoting activity as its expression is correlated with the poor clinical outcome in cancer patients.^63^ Thus, Cav1 is able to transmit both, growth inhibitory and survival promoting signals. Its role as tumor suppressor was attributed to the facts that Cav1 can inhibit cell growth^64^ and that its locus on the human chromosome 7q31.1 is frequently deleted in human cancers.^65^ However, the upregulated expression of Cav1 in epithelial tumor cells was clearly associated with advanced prostate cancer.^26, 66^ Interestingly, higher microvessel density values were found in Cav1-positive compared with the Cav1-negative human tumor specimen obtained by radical prostatectomy.^38^ In addition, pathologic angiogenesis induced by Cav1 in prostate cancer-bearing mice correlated with an increased frequency, number and size of lung metastases.^37^ Thus, apart from its role as a pro-survival protein in epithelial cancer cells, Cav1 may also function as a proangiogenic factor promoting metastatic spread in advanced prostate cancer after secretion from the tumor cells.

Consistent with earlier findings we demonstrate that the increased expression of Cav1 in epithelial cancer cells of advanced human prostate cancer tissue specimen was paralleled by a reduction of Cav1 in the tumor stroma, which is well known to have a more reactive phenotype in advanced prostate carcinoma.^67, 68, 69^ However, here we show for the first time that alterations in stromal Cav1 levels did not include the tumor vasculature. Independent of the Gleason score Cav1 was highly expressed in tumor ECs in all tissue specimen investigated. This suggests that Cav1 regulates various aspects of prostate cancer progression that contribute to treatment failure and may therefore constitute a valuable therapeutic target. Of note, recent investigations revealed that blocking the secreted Cav1 by polyclonal antibodies inhibited the growth of experimental prostate tumors in mice.^70^ Nevertheless, the accelerated growth of the untreated prostate tumors in the Cav1-deficient background hints to a potential risk of treatment strategies targeting endothelial Cav1 for improving the radiation response in these tumors. Though increased tumor growth in Cav1 knockout mice is expected be rather a long-term effect of Cav1-deficiency, these observations make a careful validation of such treatment strategies with respect to adverse growth promoting effects absolutely necessary. Further work is required to elucidate whether Cav1-dependent resistance-promoting signals from ECs can be separated from Cav1-dependent stromal signals that restrict tumor growth and may thus allow a safer targeting of Cav1 mediated radiation resistance.

Taken together our data highlight a role of stromal Cav1 for the radiation response of prostate tumors. High Cav1 expression in ECs protected experimental MPR31-4 tumors from microvascular damage, a critical component in the tumor response to radiotherapy. This is of particular interest since work from other groups already demonstrated that high Cav1 expression is associated with epithelial tumor cell radioresistance at least in pancreatic and lymphoblastoid tumor cells.^41, 42, 43^ Thus, Cav1 might be a promising target for new combinatorial therapies to overcome therapy resistance by sensitizing both, radioresistant tumor cells and the radioresistant tumor vasculature, to the cytotoxic effects of ionizing radiation. Further studies are needed to address the role of Cav1 for radiosensitivity of prostate cancer epithelial cells and to develop and validate the use of pharmacological strategies to reduce Cav1 expression or inhibit resistance-promoting Cav1-dependent signals, respectively.

## Materials and Methods

### Reagents and antibodies

Antibodies against alpha smooth muscle actin (ACTA2), Ki67 and Cav1 were from Santa Cruz (Santa Cruz, CA, USA); antibody against CD31 was from Dianova (Hamburg, Germany), against HIF1-alpha from Cayman Chemical Company (Ann Arbor MI, USA) and against Casp3 cleaved from New England Biolabs (Frankfurt, Germany). Peroxidase and fluorescently-labeled secondary antibodies were from Jackson IR Laboratories (West Grove, PA, USA).

### Human tumor tissue

Tissues from human prostate carcinomas were obtained during surgery according to the local ethical and biohazard regulations. Resected tissue specimens were processed for pathological diagnostic routine in agreement with the institutional standards and diagnoses were made based on the current WHO (World Health Organization) and the updated ISUP criteria.^4^ The local ethical review committee of the University Hospital Essen approved all the studies including human tissue specimen.

### Cells

The mouse prostate epithelial cell line MPR31-4 was a kind gift of TC Thompson, Scott Department of Urology, Baylor College of Medicine, Houston, TX, USA).^71^ Cells were cultured in Dulbecco's modified Eagle's medium with 10% fetal calf serum, 100 IU/ml penicillin and streptomycin in a humidified atmosphere (5% CO_2_ at 37 °C) and passaged twice a week. The human microvascular endothelial cell line AS-M5 was cultured in ECG medium (PromoCell).^72^

### Cav1 silencing in AS-M5 ECs

Levels of Cav1 mRNA level were downregulated by shRNA technology.^43^ Therefore a lentivirus-based vector pLentilox3.7 for mammalian expression was used. Sense and antisense oligonucleotides to Cav1 were annealed and cloned into HpaI and XhoI sites of the pLL3.7 vector directly under the control of the U6 promoter. This vector co-expresses enhanced GFP (green fluorescent protein) as a reporter gene. Lentiviral vectors were produced and titrated as described by Maier *et al.*^73^ Within 2–5 days after transduction eGFP-positive cells were sorted in a FACS Vantage cell sorter (BD Biosciences, Heidelberg, Germany).

### Flow cytometry analyses

For quantification of apoptotic DNA-fragmentation (sub-G1 population), cells were incubated for 60 min with a staining solution containing 0.1% (w/v) sodium citrate, 50 μg/ml propidium iodide, and 0.05% (v/v) Triton X-100 (v/v) and subsequently analyzed by flow cytometry (FACS Calibur, Becton Dickinson, Heidelberg, Germany; FL-2) as described elsewhere.^74^

### Cell viability assay

The number of living cells was determined upon staining of the cells with the vital dye trypan blue. For this, cells were harvested with Trypsin-ethylenediaminetetraacetic acid, resuspended in fresh medium, diluted with trypan blue and counted employing a Neubauer chamber.

### Colony formation assay

For this long-term assay, 200–1600 cells/well were plated in six-well plates. Radiation with indicated doses was performed using the Isovolt-320-X-ray machine (Seifert-Pantak, East Haven, CT, USA) at 320 kV, 10 mA with a 1.65-mm aluminum filter and a distance of about 500 mm to the object being irradiated. The effective photon energy was about 90 kV and the dose rate about 3 Gy/min. Plates were incubated for a total of 10 days to allow growth of single colonies. Cells were then fixed in 3.7% formaldehyde and 70% ethanol and subsequently stained with 0.05% Coomassie Brilliant Blue. Colonies (⩾50 cells/colony) were counted under the microscope at fivefold magnification. The survival curves were established by plotting the log of the surviving fraction against the treatment dose.

### Mouse tumor model

Cav1-deficient mice (C57Bl/6 background) and wild-type littermates were used.^44^ Mouse xenograft tumors were generated by subcutaneous injection of 0.5 × 10^6^ cells/ml MPR cells onto the hind limb of the mice. For this, MPR cells were harvested by a brief trypsinization (0.05% trypsin/0.02% ethylenediaminetetraacetic acid), washed and resuspended in phosphate-buffered saline at a density of 1 × 10^7^ cells/ml. Up to 20 animals of each experimental group received a single subcutaneous injection of 0.5 × 10^6^ viable tumor cells. For radiation therapy mice were anesthetized (2% isoflurane) and tumors were exposed to a single dose of 20 Gy using a ^60^Co-source (dose rate: 0.5 Gy/min) when tumors reached a volume about 100 mm^3^ (day 3–5 after tumor induction) or were left non-irradiated (controls). Five to twenty days later animals were sacrificed and lungs were subjected to immunohistochemistry, RNA or protein isolation. Mouse experiments followed protocols approved by the local Animal Care Committee (Regierungspräsidium Düsseldorf Az. 8.87-50.10.37.09.187; Az. 8.87–51.04.20.09.390).

### Immunohistochemistry and immunofluorescence

Paraffin-embedded tissue sections were hydrated using a descending alcohol series, incubated for 10–20 min in target retrieval solution (Dako, Glostrup, Denmark) and incubated with blocking solution (2% fetal calf serum/phosphate-buffered saline ). After permeabilization, sections were incubated with primary antibodies over night at 4 °C. Antigen was detected with a peroxidase-conjugated secondary antibody (1/250) and DAB staining (Dako). Nuclei were counterstained using hematoxylin. For immunofluorescence analysis, the antigen was detected with an anti-rabbit-Alexa488 and anti-rat-Alexa555-conjugated secondary antibody (1/500). Hoechst 33342 (Invitrogen, Karlsruhe, Germany) was used for nuclei staining. Specimens were analyzed by confocal microscopy.

### Western blot

Whole-cell lysates were generated by scraping cells into ice-cold RIPA-P buffer (150 mmol/l NaCl, 1% NP40, 0.5% sodium-desoxycholate, 0.1% sodium-dodecylsulfate, 50 mmol/l Tris/HCL pH 8, 10 mmol/l NaF, 1 mmol/l Na_3_VO_4_, supplemented with complete Protease-Inhibitor-Cocktail (Hoffmann-La Roche, Basel, Switzerland) and performing 2–3 freeze–thaw cycles. Protein samples (50–100 μg total protein) were subjected to SDS-PAGE electrophoresis and western blots were done as previously described using Cav1 (1/5000), Casp3 cl (1/1000), ACTA2 (1/200) or beta-actin (1/5000) antibodies.^75^

### Electron microscopy

Tumor tissues were fixed with cacodylate buffered glutaraldehyde 2.5%, contrasted with 1% osmium tetroxide and 1% uranyl acetate and embedded in EPON. Ultra-thin sections were mounted on 200-mesh hexagonal cooper grids. For contrast enhancement uranyl acetate and lead citrate were applied. Sections were analyzed using a Zeiss transmission electron microscope (Carl Zeiss Microscopy, Oberkochen, Germany) (EM 902 A) at 80 KV. Digital image acquisition was performed on a Morula slow-scan-CCD camera (Tietz Video and Image Processing, Gauting, Germany) connected to a PC running ITEM 5.0 (ITEM Software, Irvine, CA, USA).

### Statistical analysis

Paired or unpaired two-tailed *t*-tests were performed using GraphPad InStat3 software (GraphPad, La Jolla, CA, USA) depending on effective matching of analyzed data. s.d. or s.e.m. were indicated by error bars. Significance was assumed for *P*-values ⩽0.05. *P⩽0.05; **P⩽0.01; ***P⩽0.005; ****P⩽0.001.
