# Supplementary material for: Endothelial Caveolin-1 regulates the radiation response of epithelial prostate tumors
Source: Oncogenesis. 2015 May 18;4(5):e148–. doi: 10.1038/oncsis.2015.9 (PMC4450264; doi:10.1038/oncsis.2015.9)
Supplement: Supplementary Information [file oncsis20159x1.doc]

**Supplemental Figures**

**Supplemental Figure S1**


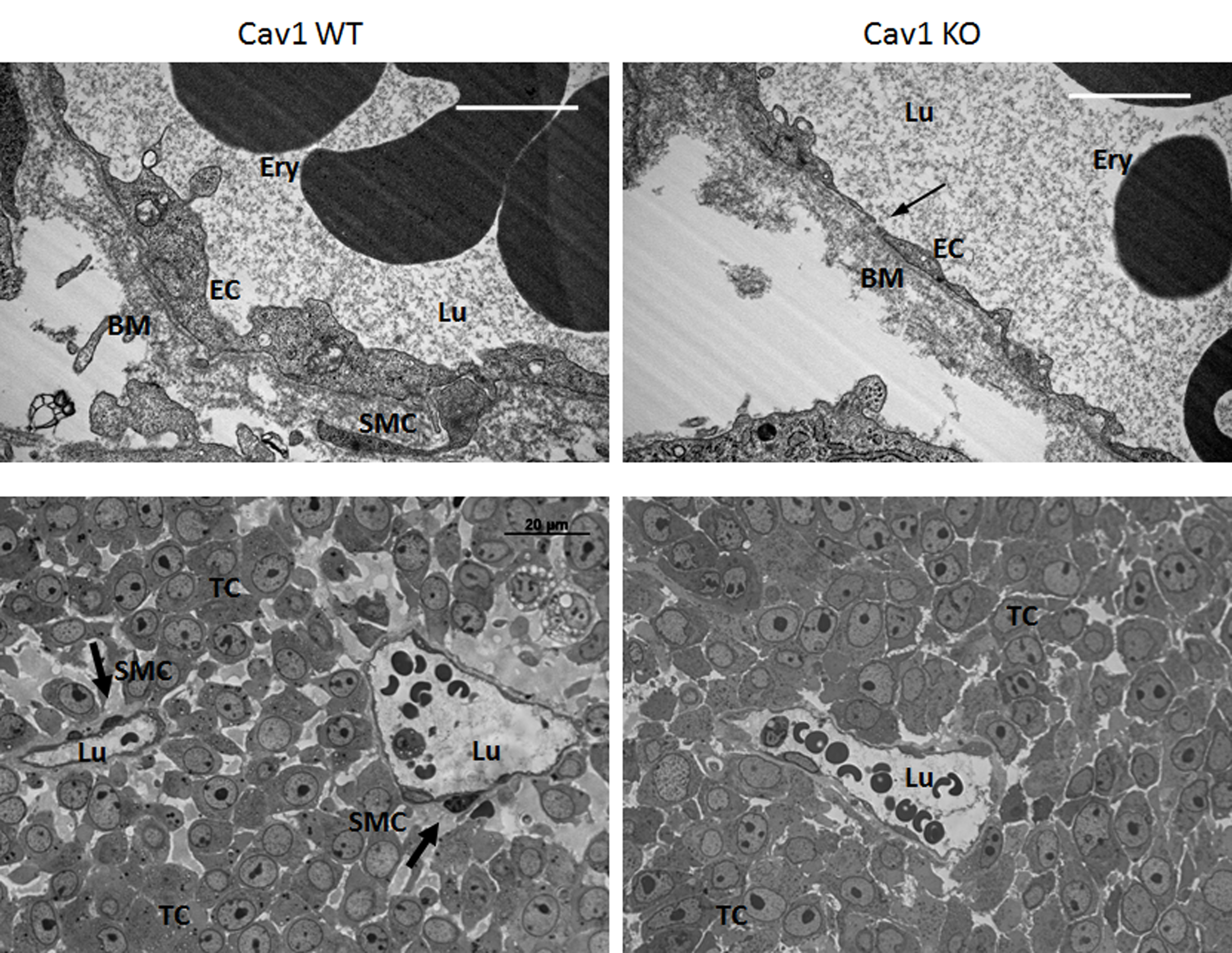


**Figure S1**

**Ultrastructural analysis of tumor samples showed an irregular lining of SMC and the presence of fenestrae in angiogenic tumor endothelial cells from tumors of Cav1-deficient animals.**

Subcutaneously grown MPR31-4tumorsof Cav1-deficient mice (KO) and wildtype littermates (WT) were removed 21-25 days after tumor induction and subjected to electron microscopic analysis (upper panel).A defective and irregular lining of angiogenic endothelial cells (EC) is emphasized by an arrow (fenestrae). Semi thin sections were analysed using phase contrast microscopy (lower panel). Association of pericytes/SMC to angiogenic tumor blood vessels are emphasized by arrows.BM basement membrane, SMC smooth muscle cell, Ery erythrocyte, Lu lumen. Representative images of 3 independent experiments are shown.Scale bar 2µm, upper panel; 20µm, lower panel).

**Supplemental Figure S2**


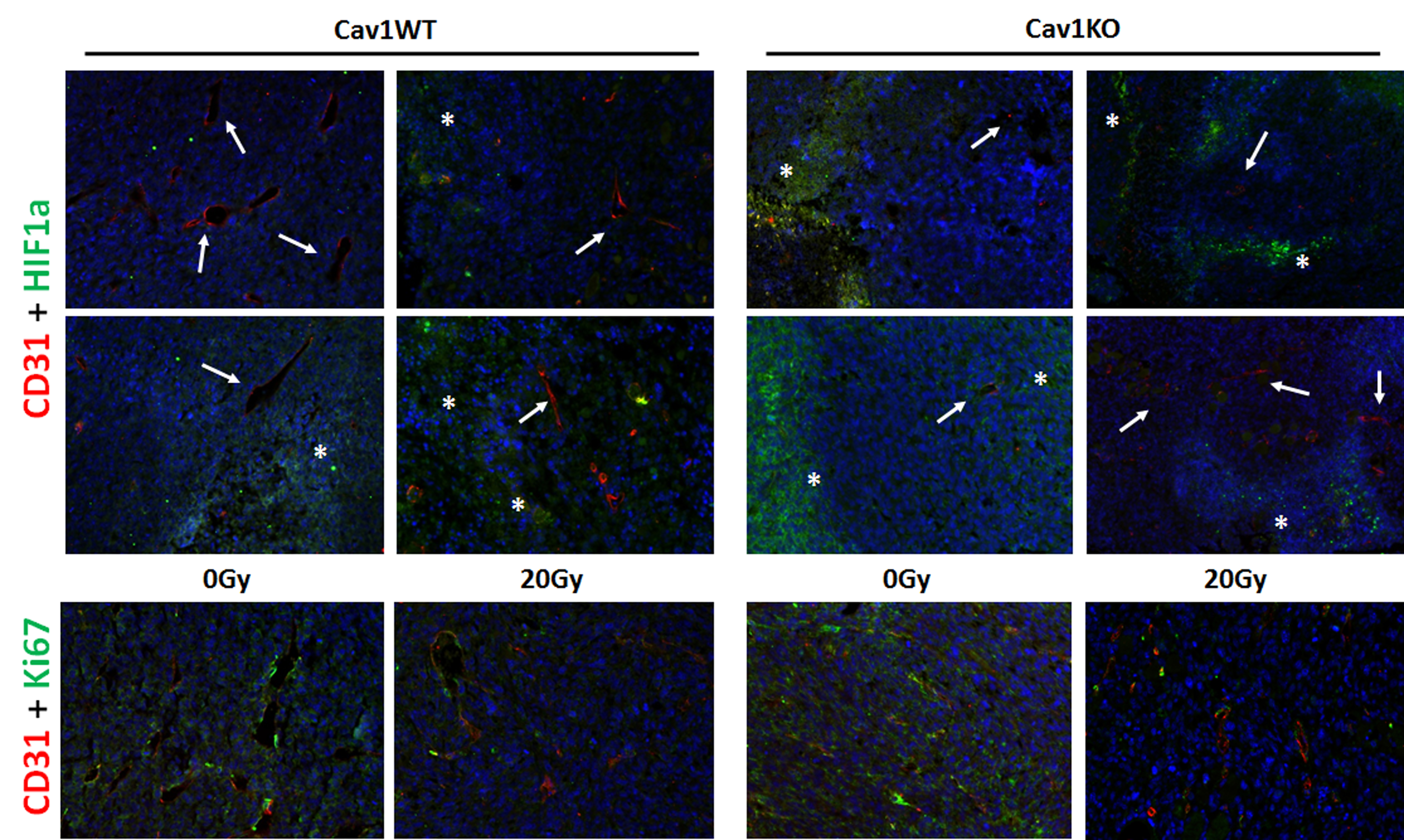


**Figure S2**

**Prostate tumors grown in Cav1-deficient mice showed increased expression of hypoxia-inducible factor 1alpha (HIF1α) as well as regions with increased cell proliferation.**

Subcutaneously transplanted MPR31-4 tumors from Cav1-deficient mice (KO) and wildtype controls (WT) were analysed by immunofluorescence at the end of the experiment when tumors reached the critical size (sham controls: day 8-10; irradiated tumors: around day 20). Sections were stained for CD31 (red), and hypoxia inducible factor 1-alpha (HIF1α) (green) or the proliferation marker Ki67 (green). Hif1α-immunoreactive regions were emphasised by asterisks; arrows point towards vascular structures. Magnification 20x.

**Supplemental Figure S3**


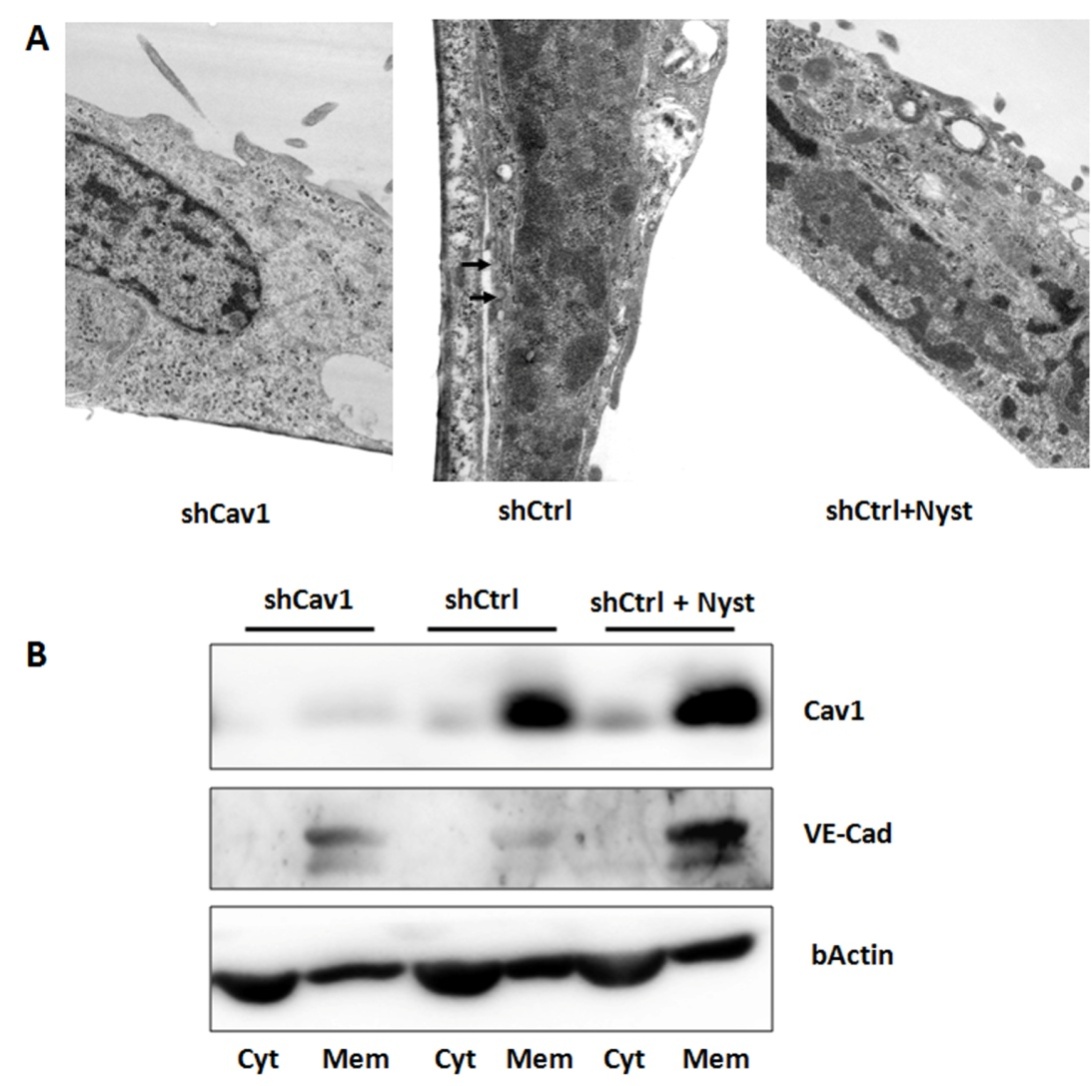


**Figure S3**

**Nystatin-treatment did neither affect Cav1 content nor its localization in cultured AS-M5 endothelial cells.**

**A** The presence of caveolae was analyzed in cultured shCav1-transfected and control-transfected (shCtrl) AS-M5 endothelial cells by ultrastructural analysisusing electron microscopy. In addition, control-transfected AS-M5 cells were treated with Nystatin (10µg/ml) in order to inhibit caveolae formation (shCtrl+Nyst). Arrows point to Caveolae. **B** Cav1 expression and membrane localization were further determined using Western blot analysis in cytosolic (Cyt) and membrane (Mem) fractions prepared from total cell lysates. VE-Cadherin expression served as control for the preparation of the membrane fractions. Representative blots from at least three independent experiments are shown.

**Supplemental Figure S4**


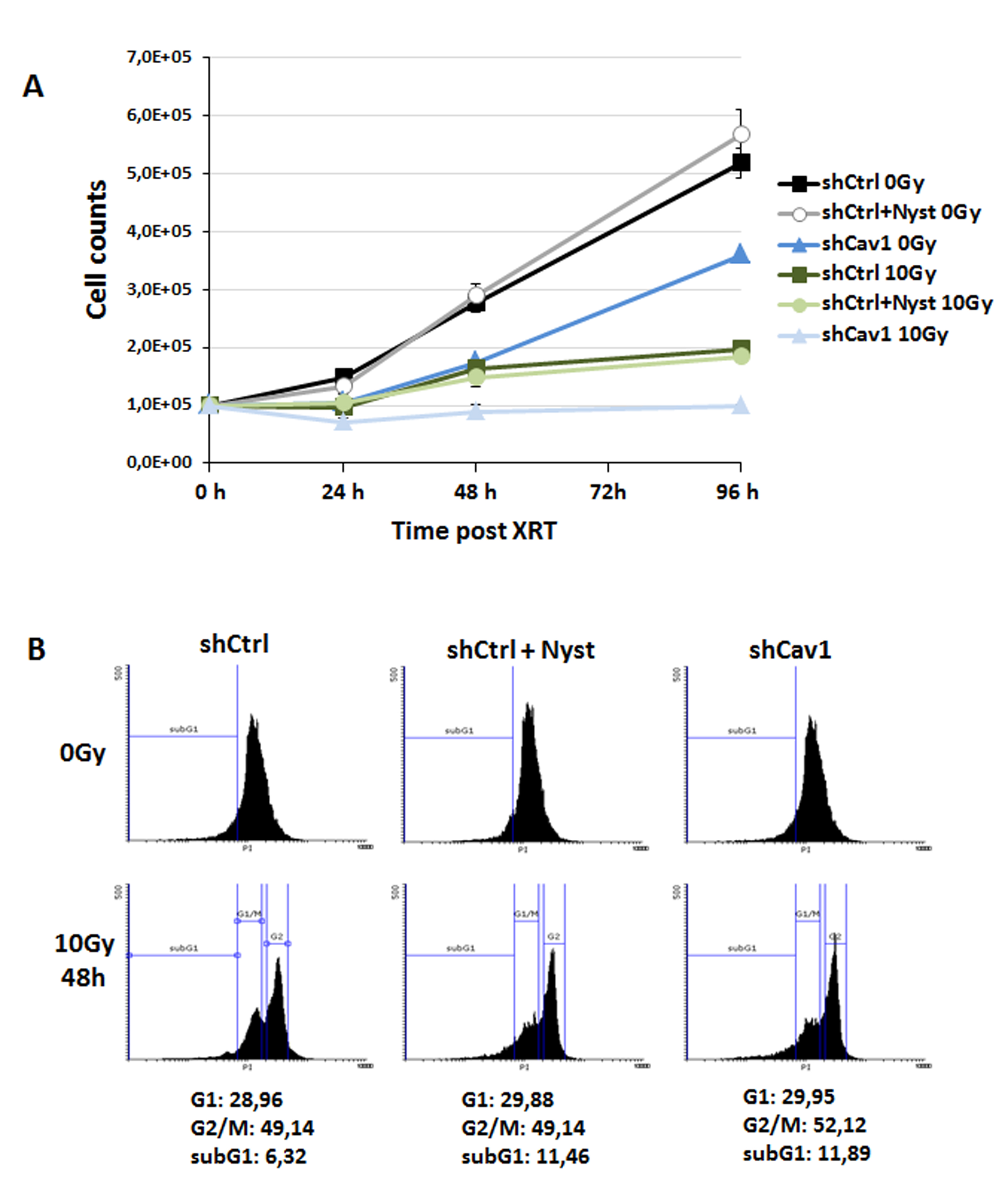


**Figure S4**

**Reduction of Cav1 levels decreases overall endothelial cell proliferation but does not affect cell cycle distribution before/after irradiation.**

**A** Cell proliferation was analyzed by cell counting in cultured shCav1-transfected and control-transfected (shCtrl) AS-M5 endothelial cells at the indicated time points after irradiation with 10Gy. In addition, control-transfected AS-M5 cells were treated with Nystatin (10µg/ml; shCtrl+Nyst). **B** Cell cycle distribution was assessed by measuring cellular DNA content 48h after exposure to 0 or 10 Gy by flow cytometry. Representative graphs from two independent experiments are shown.
